# Supplementary figures and images for: A kinetic model of the central carbon metabolism for acrylic acid production in Escherichia coli
Source: PLoS Comput Biol. 2021 Mar 8;17(3):e1008704. doi: 10.1371/journal.pcbi.1008704 (PMC7971886; doi:10.1371/journal.pcbi.1008704)

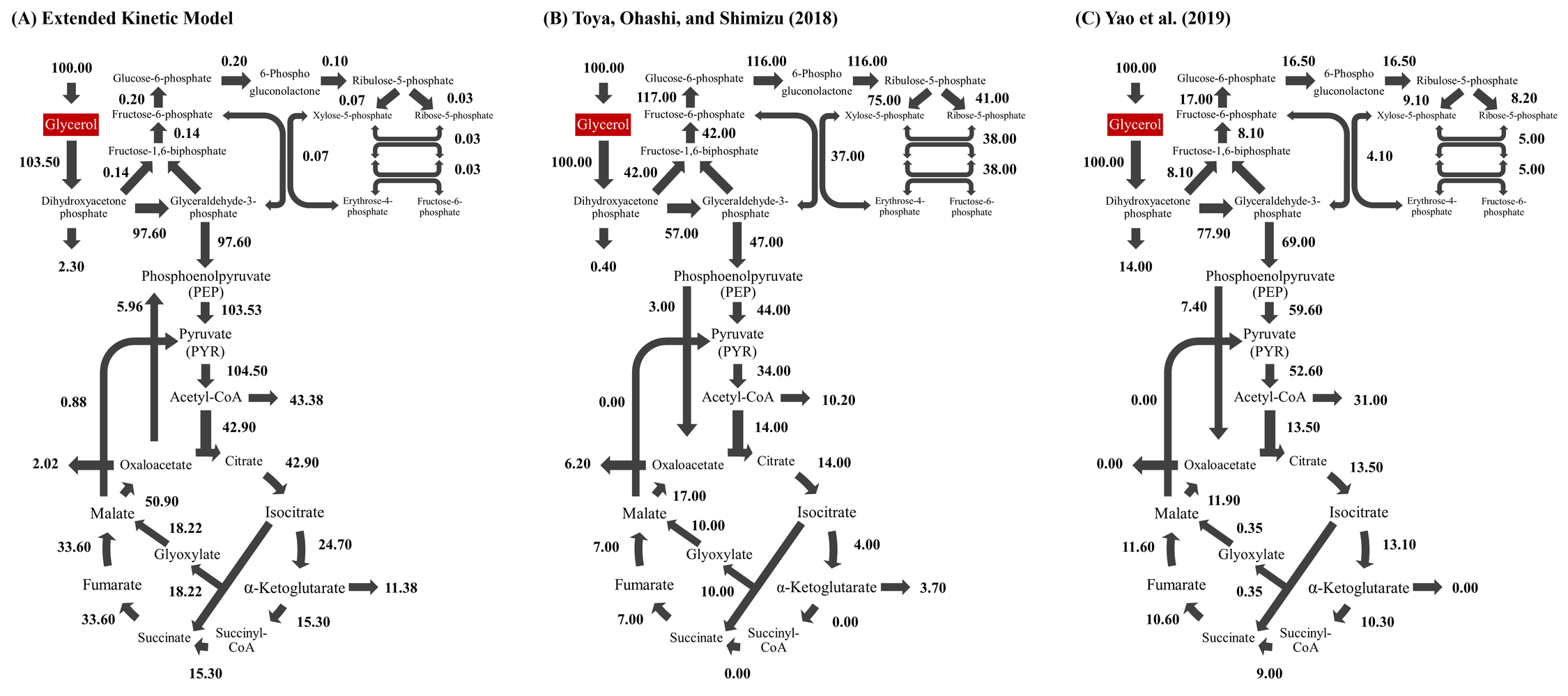

Supplement: S1 Fig — (A) Steady-State flux distribution from glycerol obtained from the extended kinetic model of E.coli’s CCM; (B) Flux distribution from glycerol obtained experimentaly by Toya et al. (2018) [12]; (C) Flux distribution from glycerol obtained experimentaly by Yao et al. (2019) [13]. (TIF) [file pcbi.1008704.s001.tif]

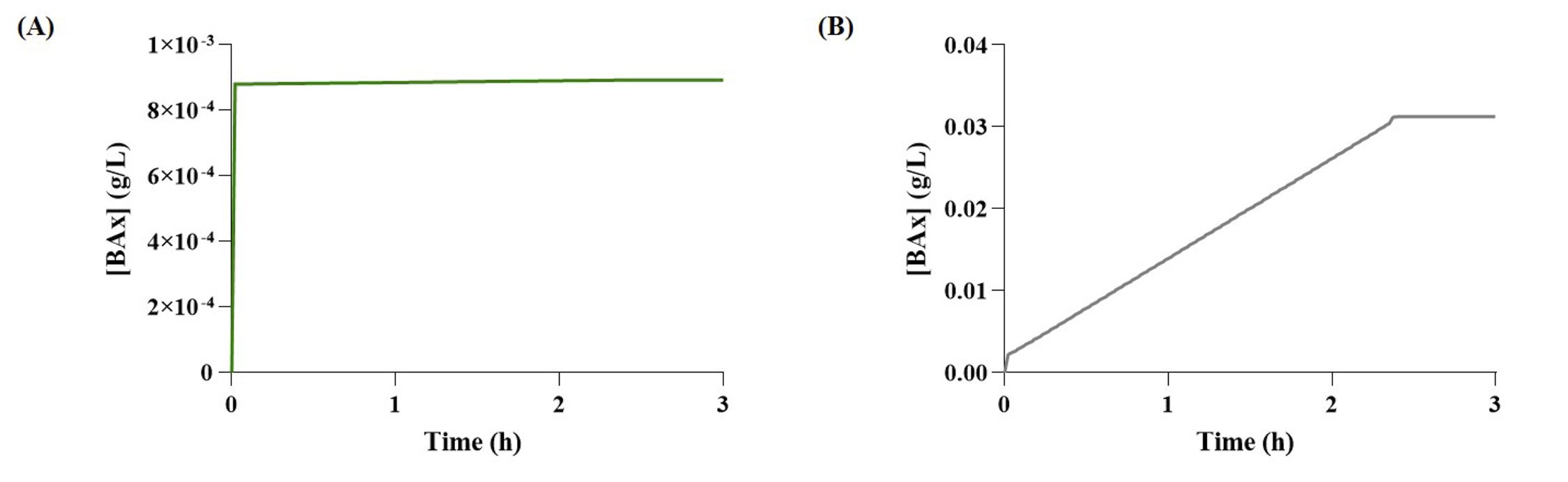

Supplement: S2 Fig — (A) β-alanine concentration using the Vmax for the aspartate carboxylase (AspC) enzyme calculated using Method 1 (1.15x10^-05^ mM/s). (B) β-alanine concentration using the Vmax for the AspC calculated using Method 2 (57 mM/s). (TIF) [file pcbi.1008704.s002.tif]

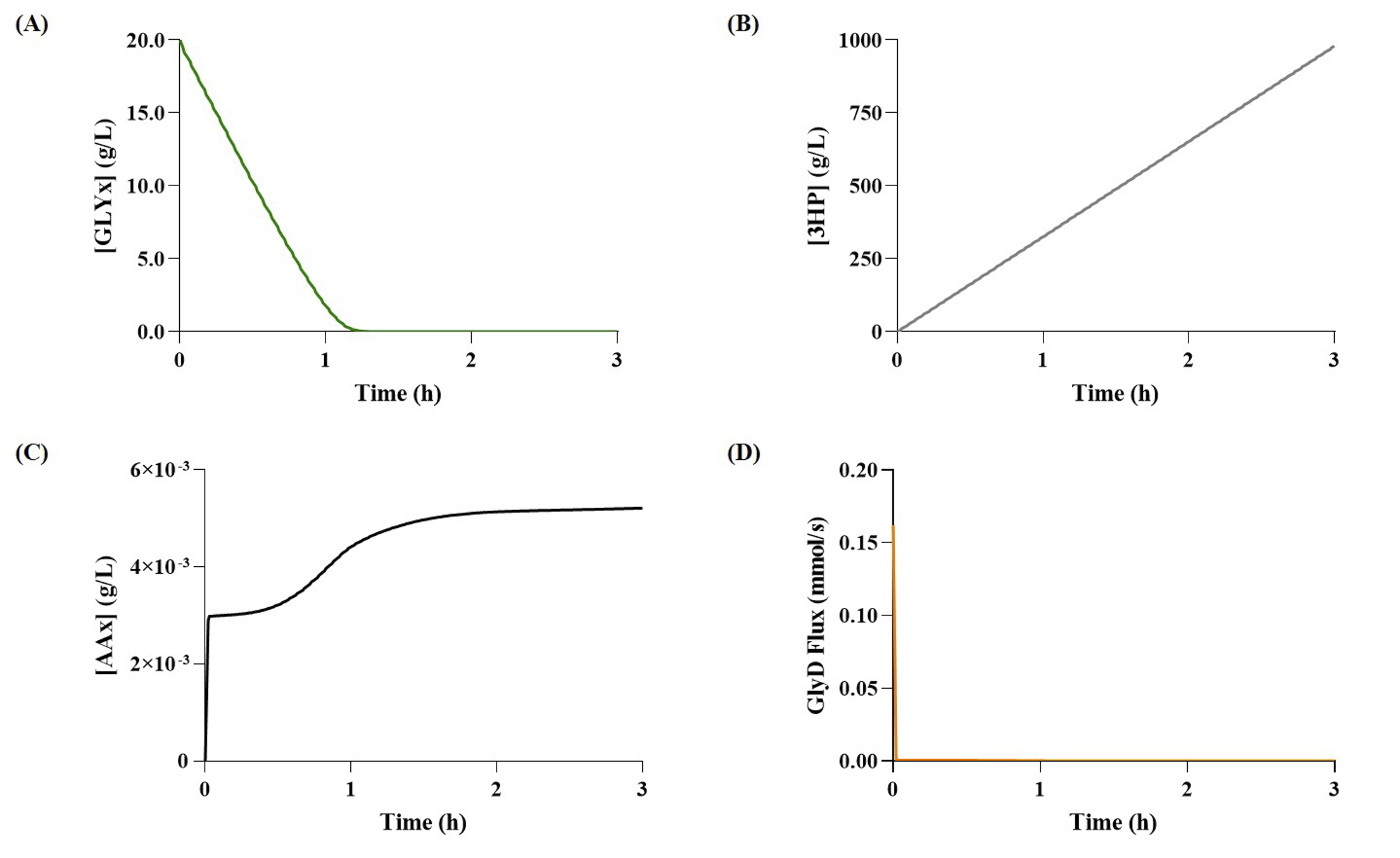

Supplement: S3 Fig — (A) Glycerol (GLY) consumption. (B) Production of 3-hydroxypropionate (3-HP). (C) Acrylic acid (AA) production. (D) Flux of the glycerol dehydrogenase (GlyD). (TIF) [file pcbi.1008704.s003.tif]

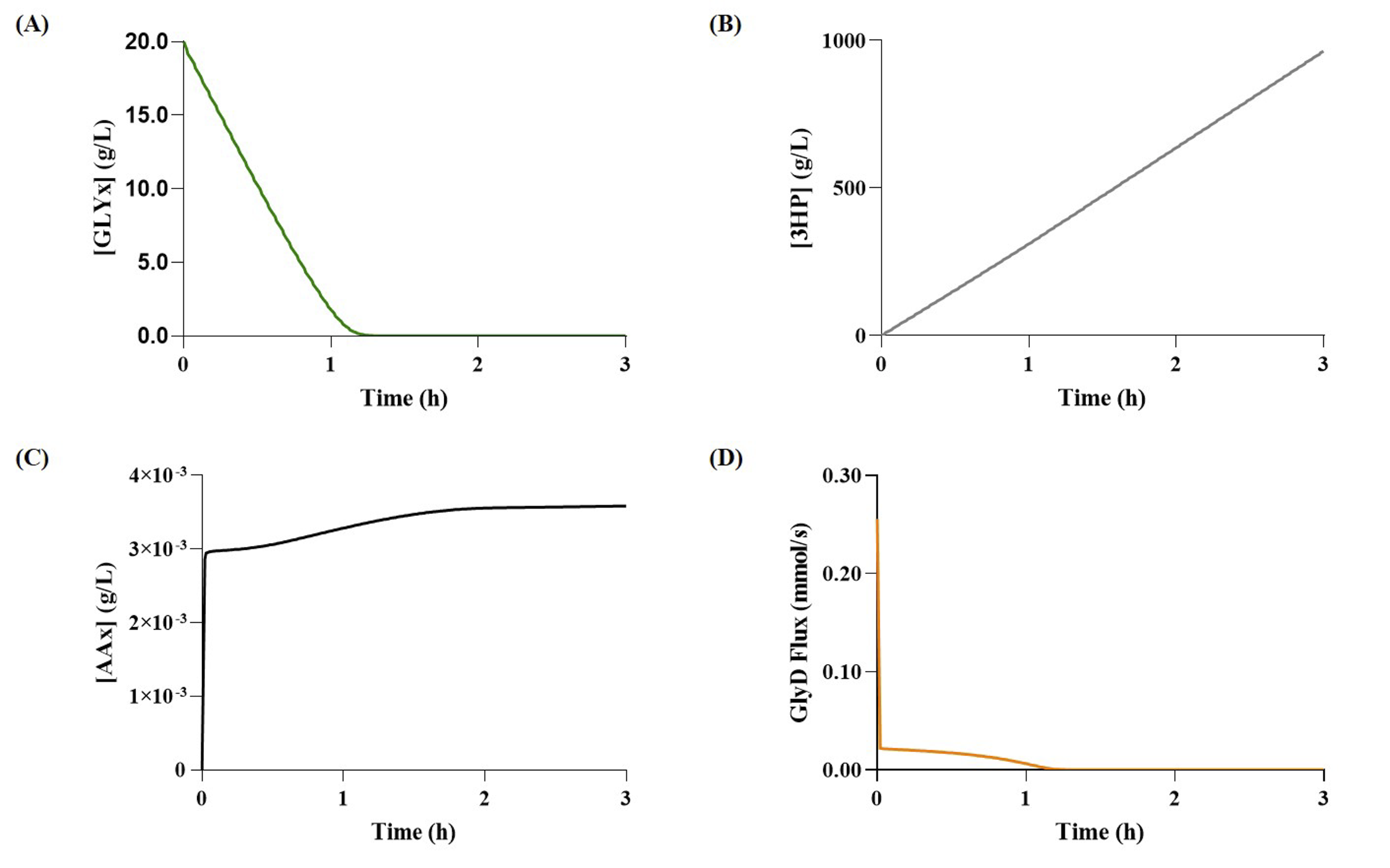

Supplement: S4 Fig — (A) Glycerol (GLY) consumption. (B) Production of 3-hydroxypropionate (3-HP). (C) Acrylic acid (AA) production. (D) Flux of the glycerol dehydrogenase (GlyD). (TIF) [file pcbi.1008704.s004.tif]

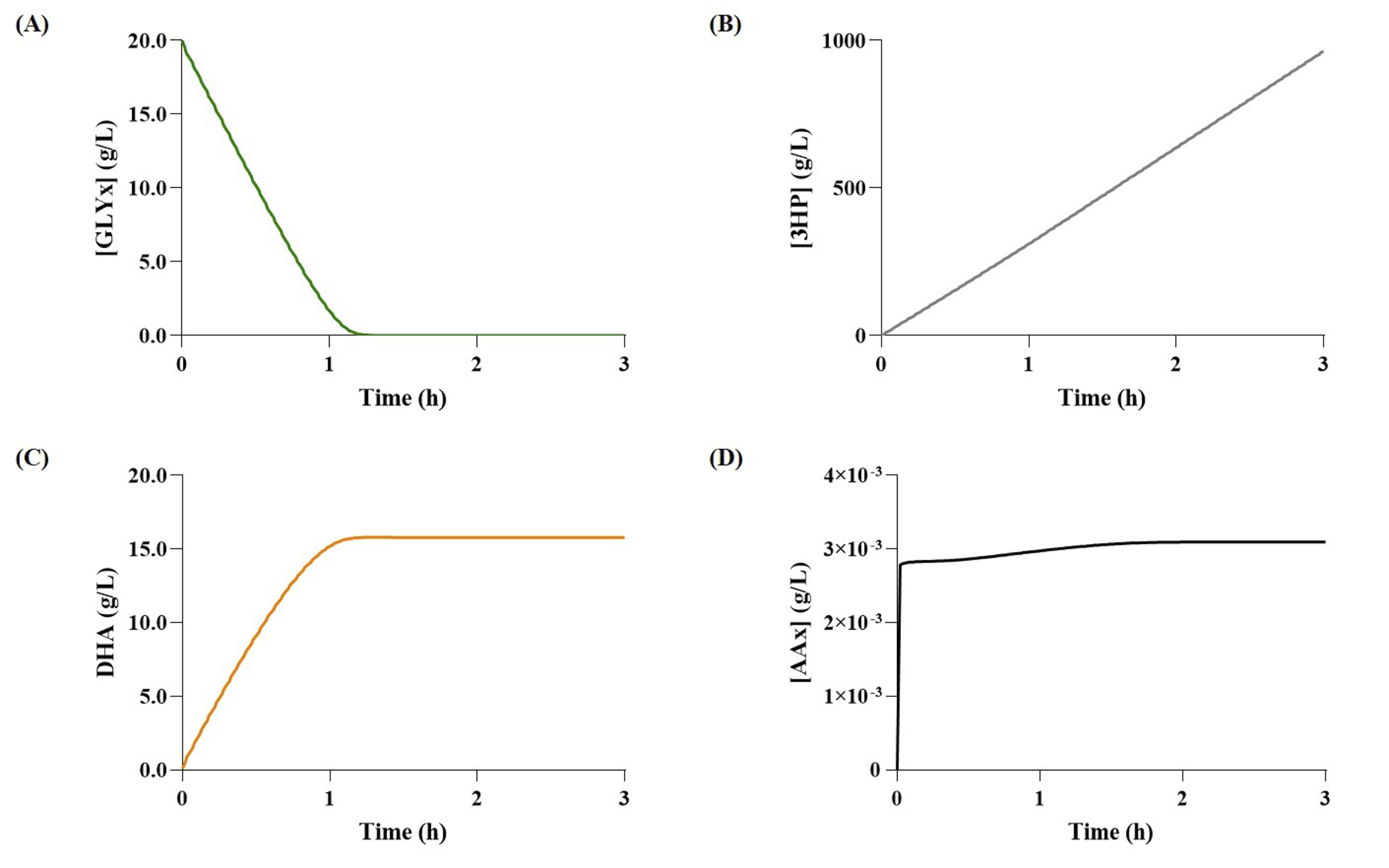

Supplement: S5 Fig — (A) Glycerol (GLY) consumption. (B) Production of 3-hydroxypropionate (3-HP). (C) Variation of dihydroxyacetone (DHA) concentration. (D) Acrylic acid (AA) production. (TIF) [file pcbi.1008704.s005.tif]

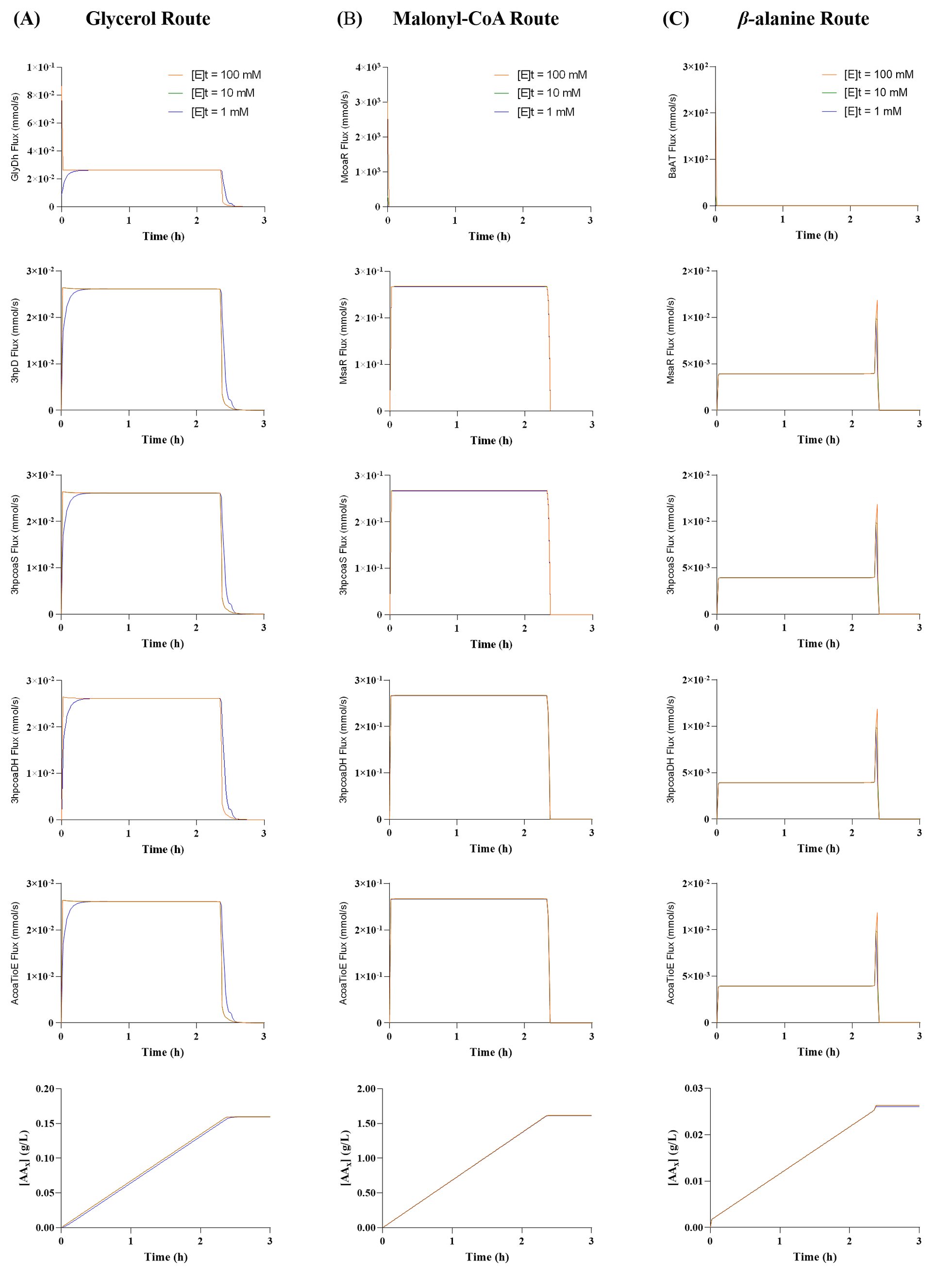

Supplement: S6 Fig — Time course simulation of AA production from glucose and fluxes of the heterologous reactions when using different enzyme concentrations to determine the Vmax value, according to method 2, for the glycerol route (A), malonyl-CoA route (B), and β-alanine route (C). Three concentrations were simulated: 100 mM (orange lines), 10 mM (green lines), and 1 mM (blue line) (TIF) [file pcbi.1008704.s006.tif]

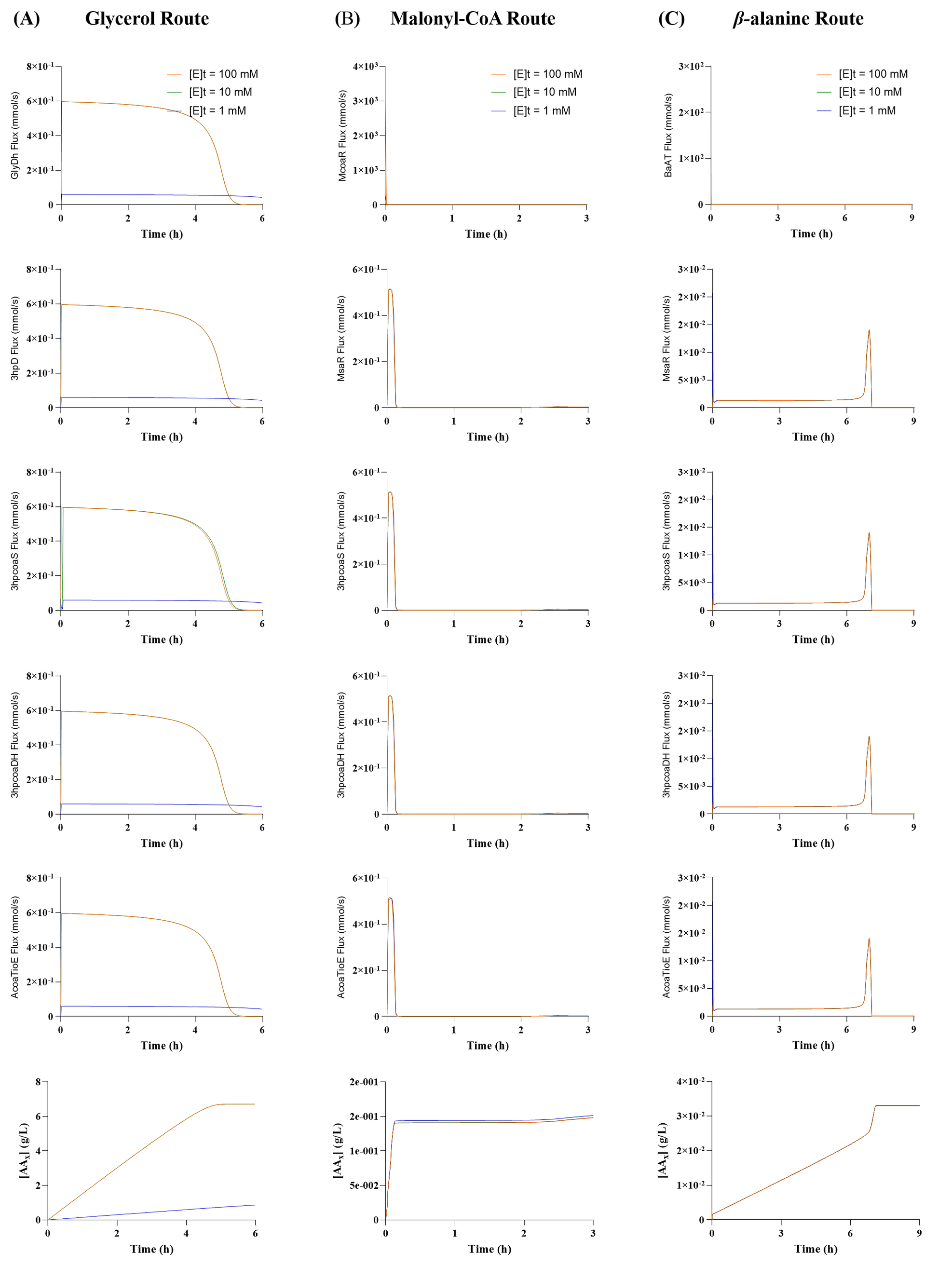

Supplement: S7 Fig — Time course simulation of AA production from glycerol and fluxes of the heterologous reactions when using different enzyme concentrations to determine the Vmax value, according to method 2, for the glycerol route (A), malonyl-CoA route (B), and β-alanine route (C). Three concentrations were simulated: 100 mM (orange lines), 10 mM (green lines), and 1 mM (blue line) (TIF) [file pcbi.1008704.s007.tif]
