# Supplementary material for: A kinetic model of the central carbon metabolism for acrylic acid production in Escherichia coli
Source: PLoS Comput Biol. 2021 Mar 8;17(3):e1008704. doi: 10.1371/journal.pcbi.1008704 (PMC7971886; doi:10.1371/journal.pcbi.1008704)
Supplement: S1 Appendix — Additional results, explanations behind parameter adjustments adopted to circumvent simulation issues, and results for the Vmax calculation using method 1 and method 2. (PDF) [file pcbi.1008704.s011.pdf]

S1 Appendix

1 Supplementary Results

1.1 Steady-State Flux Distribution

The original kinetic model from Millard et al. (2016) [21] was developed and validated for *E.coli* grown in glucose. Nevertheless, this work aims to also analyse AA production from glycerol. The carbon provided by this metabolite enters the glycolysis as dihydroxyacetone phosphate (S1 Fig), significantly changing the flux distribution. Hence, the predicted steady-state flux distribution of the model was compared with experimentaly determined values, since, theoretically, the more similar the results, the strongher the predictions. However, significant differences emerged. The major one is that, according to the experimental results, more flux should enter the gluconeogenese pathway. Unfortunatly, the model did not replicate that as only 0.14 percent of the provided carbon is directed towards fructose-6-phosphate and 97.6 percent towards phosphoenolpyruvate (S1 Fig A), compared to the 42 and 57 percent, respectively, measured by Toya et al. [12] (S1 Fig B), and 8.1 and 69 percent from Yao et al. [13] (S1 Fig C). Consequentialy, the remaining fluxes diverged as the model predicted a lower flux in the pentose-phosphate pathway, and, on the other hand, a three times higher flux in the citric acid cycle (S1 Fig). This had a significant impact in the prediction of the models, specially for the Gly_Mcoa and Gly_Ba models since they are the most affected by the difference in TCA cycle flux. However, this should not have a major impact in the Gly_Gly model as there are no significant differences in the glycerol uptake and consumption flux.

1.2 Simulation Problems

During the simulations, several issues arised. The first one was related to the V_{max} of the aspartate carboxylase (AspC) enzyme during the CCM extension. The second problem was related by the assimilation of glycerol to the CCM in the glycerol and malonyl-CoA models when using glycerol as carbon source, from which it was only possible to circumvent the first one. In this section these problems will be further explained as well as the methods used to resolve them.

1.2.1 Extension of the Central Carbon Metabolism (CCM)

Even though the simulation of glycerol and malonyl-CoA production was successful, the production of β -alanine exhibited a major setback. The extremely low V_{max} of the AspC enzyme was limiting the amount of β-alanine produced (S2 Fig A). The V_{max} was calculated with the flux obtained from the stoichiometric model simulation (Method 1), which resulted in a relevant bottleneck. Thus, this parameter was recalculated using the method developed for the heterologous pathway reactions parameters (Method 2), shifting the β-alanine flux production limits towards L-aspartate formation. According to Ramjee et al. (1997) [52], the K_{cat} value for this enzyme was 0.57 s^{-1}. Therefore, the V_{max} value was set to 57 mM/s in all the models, which drastically altered the production of β-alanine (S2 Fig B).

1.2.2 Glycerol Model

Issues were found in the models created for modeling 3-HP and AA production, when glycerol was used as carbon source. The carbon flow toward the CCM stopped seconds after the simulation started, leading to the accumulation of 3-HP (S3 Fig). After analyzing the system, it was determined that two factors were responsible for this behavior. The first factor is associated with the heterologous pathway, as the V_{max} of all reactions in this pathway was set as not to limit flux through these reactions. Therefore, these reactions would uptake most of the available glycerol, thus limiting the amount of carbon towards

the CCM, which eventually led to the depletion of crucial cofactors for AA synthesis, such as NAD^+ and ATP. The second factor is associated with the NAD^+ affinity to enzymes glycerol dehydrogenase (GlyD) and 3-hydroxypropionaldehyde dehydrogenase (3hpaD). The K_m value in the GlyD is over tenfold higher than in 3hpaD, thus the low concentration of NAD^+ available was mainly used by the 3hpaD, which blocked the flux towards the CCM, exacerbating the energy production problem even further.

A couple of hypotheses were devised to circumvent these problems. Because the NAD^+ affinity issue was blocking the flux of carbon towards the CCM, the first hypothesis was increasing the affinity of the GlyD enzyme towards NAD^+. When comparing the affinity with other enzymes present in the model, this enzyme had a significantly higher value. Therefore, other works that characterized this enzyme were searched, to find an alternative for this K_m value. In the work of Zhang et al. (2010) [55] this enzyme, using another substrate, was described with an affinity towards NAD^+ of 0.0165 mM. Thus, the value was updated and simulated in a period of three hours. However, the problem persisted as most glycerol was still going towards the formation of 3-HP (S4 Fig).

The next strategy was increasing the maximal rate of the GlyD to increase the flux in the CCM, allowing the continuous production of AA. Hence, the V_{max} was recalculated according to Method 2, resulting in a value of 4298.4 mM/s. However, even then the majority of glycerol provided was directed towards the heterologous pathway (S5 Fig B), and the small amount that was directed towards the CCM was accumulated as dihydroxyacetone because the activity of the dihydroxyacetone phosphate transferase (DhaPT) was not enough to consume that metabolite (S5 Fig C).

The third hypothesis involved decreasing the affinity of the 3hpaD towards NAD^+, to improve the activity of the GlyD, and the maximum rate of the glycerol dehydratase (GlyDH) to control the flux that is directed towards to heterologous route. Since no further K_m value for the 3hpaD was found in the

literature, it was assumed that this enzyme had a similar affinity as the GlyD (K_m = 0.8 mM). Furthermore, the V_{max} of the GlyDH was set to 0.621 mM/s, representing an enzyme concentration of 10 mM instead of the 100 mM used in Method 2. These changes allowed directing the flux towards the CCM, which resulted in the production of AA without the excessive accumulation of any intermediaries (Fig 3).

1.3 Enzyme Concentration to determine the V_{max} value

The V_{max} of the heterologous reactions was determined according to equation (2). However, since the enzyme concentation when the turnover was determined was not available, an excessive value of 100 mM was selected, similarly to what Machado et al. (2014) [31] used. Even though this value is significantly over biologicaly possible, the goal was to avoid creating bottlenecks in the model that would impair the analysis performed by this work. Nevertheless, concentrations of 10 and 1 mM, were tested to assess if the lower concentrations would impact AA production. To do so we compared AA production and the flux os the heterologous reactions in the three pathways for the AA producing models.

Starting with an enzyme concentration of 10 mM, no significant changes were observed using either glucose or glycerol as carbon source. Even though the flux of the heterologous reactions slightly changed, especially for the glycerol route using glucose (S6 Fig A), the final AA concentration obtained with 10 mM (S6 Fig and S7 Fig) was not significantly different when comparing to the results with 100 mM of enzyme (Fig 3, Fig 5 and Fig 7). This means that this concentration did not create bottlenecks within the model, and thus could be used for the enzyme concentration. On the other hand, changing the value to 1 mM impacted the flux of the heterologous reactions in the Gly_Gly model, causing a significant reduction of AA production (S7 Fig A). Even though the results of the remaining models were not significantly impacted by the lower concentration (S6 Fig and S7 Fig), because it impaired one model, it could not be used to determine the V_{max} values.

This analysis concluded from the three tested values, 100 mM and 10 mM could both be used for the enzyme concentration. Nevertheless, the selected value was 100 mM since this value was already established in the literature [31].
