# Supplementary material for: A kinetic model of the central carbon metabolism for acrylic acid production in Escherichia coli
Source: PLoS Comput Biol. 2021 Mar 8;17(3):e1008704. doi: 10.1371/journal.pcbi.1008704 (PMC7971886; doi:10.1371/journal.pcbi.1008704)
Supplement: S2 Appendix — This file presents all the kinetic parameters and equations used to model AA production. (PDF) [file pcbi.1008704.s012.pdf]

S2 Appendix

1 Kinetic Data

In this section it is laid out the kinetic information found in a variety of databases for all the reactions that needed to be included in the model. First are described the reactions for the central carbon metabolism (CCM) extension, then the heterologous reactions required to produce acrylic acid (AA), and finally the transport reactions added to transport end metabolites to the extracellular compartment.

1.1 Extension of the Central Carbon Metabolism (CCM)

The first step to produce AA is to extend the CCM to include the production of glycerol, malonyl-CoA, and *β*-alanine, which are crucial intermediary metabolites for the heterologous pathways and naturally produced in *Escherichia coli*. Nine reactions were required to obtain those metabolites. For the glycerol case, two enzymes allow to produce glycerol and are catalyzed by the glycerol-3-phosphate dehydrogenase (G3pD) and the glycerol-3-phosphate phosphatase (G3pP), and three allow the utilization of glycerol as a carbon source, catalyzed by the glycerol kinase (GlyK), glycerol dehydrogenase (GlyD) and the dihydroxyacetone phosphate transferase (DhaPT). To produce malonyl-CoA, only one reaction was added, and it is catalyzed by the acetyl-CoA carboxylase (AccC). Finally, the remaining three reactions, catalyzed by the aspartate aminotransferase (AspAT), the aspartate carboxylase (AspC), and the *L*-glutamate dehydrogenase (GluD), are required to produce *β*-alanine.

1.1.1 *Glycerol-3-phosphate dehydrogenase (G3pD)*

This enzyme is responsible for the conversion of dihydroxyacetone phosphate to glycerol-3-phosphate, coupled with the oxidation of NADPH to $NADP^+$. Three works were found regarding this

enzyme [32,33,34] The kinetic properties of G3pD were fully characterized in the work of Edgar and Bell (1978) [32]. They reported a K_m of 0.18 mM for dihydroxyacetone phosphate ($K_{m,a}$), 0.0034 mM for NADPH ($K_{m,b}$), 0.03 mM for glycerol-3-phosphate ($K_{m,p}$), and finally 0.165 mM for $NADP^+$ ($K_{m,q}$) and a K_{eq} of 900 for this reaction. They did not accurately identify the kinetic mechanism of the enzyme, however they stated that the most probable one was the Rapid Equilibrium Random Bi Bi. Later that year, the same authors published another work concerning the kinetic properties of this enzyme where they reported two more K_m values for NADPH, 0.0041 mM and 0.0037 mM [33]. Moreover, a characterization made by Kito and Pizer (1969) [34] reported K_m values for dihydroxyacetone phosphate and glycerol-3-phosphate of 0.17 mM and 0.21 mM, respectively. This combination of parameters was used to calculate the mean values that were reported in Table 5.

1.1.2 *Glycerol-3-phosphate phosphatase (G3pP)*

The G3pP is responsible for the production of glycerol from glycerol-3-phosphate. For this enzyme, only the work of Salles et al. (2007) [35] was considered. This enzyme follows a single substrate Michaelis-Menten kinetic, and according to their work, the respective K_m for glycerol-3-phosphate was equal to 2.9 mM, as shown in Table 5.

1.1.3 *Glycerol-3-phosphate phosphatase (G3pP)*

This enzyme uses the dephosphorylation of ATP to convert glycerol into glycerol-3-phosphate. The kinetic parameters of this enzyme were found in different works. Pettigrew et al. (1990) [36] reported a K_m for glycerol ($K_{m,a}$) of 0.0049 mM and for ATP ($K_{m,b}$) of 0.0084 mM, and a dissociation constant for ATP ($K_{d,a}$) of 0.086 mM, and they also reported that the enzyme seemed to have a random Bi Bi kinetic mechanism. In turn, Hayashi and Lin (1967) [37] reported a $K_{m,a}$ of 0.0013 mM, Thorner and

Paulus (1973) [38] reported a $K_{m,a}$ of 0.01 mM, and finally, the work from Applebee et al. (2011) [39] reported a $K_{m,b}$ of 0.0078 mM. The resulting mean values for each parameter are presented in Table 5.

1.1.4 *Glycerol dehydrogenase (GlyD)*

The GlyD is the enzyme responsible for the reversible conversion of glycerol into dihydroxyacetone, coupled with the reduction of NAD^+ to NADH. Since it was not possible to determine the missing V_{max} value for the reverse reaction, the GlyD was included as an irreversible reaction. This reaction was characterized in *E. coli* by Piattoni et al. (2013) [54]. In their work they presented six different K_m values for Glycerol ($K_{m,a}$), 76, 81,47, 46, 13 and 24 mM, and for NAD^+ ($K_{m,b}$), 0.81, 1, 3.2, 0.8, 1.4 and 1.1 mM. Moreover, according to the authors, this enzyme exhibited a behavior that seemed to fit hill cooperativity kinetics, and thus they also proceeded to report six values for the hill coefficient (n) of 0.9, 0.9, 1, 1,1.1 and 1. The resulting mean values for each parameter are presented in Table 5.

1.1.5 *Dihydroxyacetone phosphate transferase (DhaPT)*

The DhaPT enzyme catalyzes the final reaction that connects glycerol to the remain of the CCM, through the conversion of dihydroxyacetone into dihydroxyacetone phosphate, an intermediary of the glycolysis pathway, coupled with the dephosphorylation of phosphoenolpyruvate to pyruvate. This enzyme was not fully characterized for both substrates, as the literature revealed a lack of K_m values for phosphoenolpyruvate in the used databases. Therefore, mass action kinetics were used to represent the dynamics of this reaction, and the k value was calculated using method 1 (S4 Table).

1.1.6 *Acetyl-CoA carboxylase (AccC)*

This enzyme is responsible for the conversion of acetyl-CoA to malonyl-CoA coupled with the dephosphorylation of ATP. The kinetic parameters of this enzyme were described by Soriano et al.

(2005) [40], in which they reported a K_m for acetyl-CoA ($K_{m,a}$) of 0.018 mM and for ATP ($K_{m,b}$) of 0.06 mM. Moreover, a study by Freiberg et al. (2004) [41] and another by Meades et al. (2010) [42], showed that malonyl-CoA, a product of this reaction, presented competitive inhibition towards acetyl-CoA, and therefore both presented values for the inhibition constant for this metabolite ($K_{i,p}$) of 0.1 mM and 0.04 mM, respectively. These parameters were used to calculate the mean values reported in Table 5.

1.1.7 *Aspartate aminotransferase (AspAT)*

The AspAT is responsible for the formation of *L*-aspartate and α-ketoglutarate from oxaloacetate and *L*-glutamate. Yagi et al. (1985) [47] presented a kinetic description of this enzyme in which K_m values of 15 mM, 0.01 mM, 0.24 mM, and 1.3 mM, were reported for *L*-glutamate ($K_{m,a}$), oxaloacetate ($K_{m,b}$), aspartate ($K_{m,p}$), and α-ketoglutarate ($K_{m,q}$), respectively. In addition, K_m values were also reported by Chow et al. (2004) [48] for α-ketoglutarate of 0.48 mM, by Mavrides and Orr (1975) [49] of 0.37 mM and 4.4 mM for oxaloacetate and *L*-aspartate respectively, by Deu et al. (2002) [50] for α-ketoglutarate of 0.59 mM and aspartate of 1.9 mM, and finally, by Fernandez et al. (2012) [51] of 37 mM and 5.2 mM for *L*-glutamate and for *L*-aspartate of 3.1 and 4 mM. Unfortunately, the underlying mechanism of this enzyme was not specified in this paper, but according to BioCyc, this enzyme is known to follow a reversible Ping-Pong Bi Bi mechanism. Moreover, to finish this enzyme characterization, the eQuilibrator database was accessed to retrieve the K_{eq}, whose value was 3.2. The mean values are presented in Table 5.

1.1.8 *Aspartate carboxylase (AspC)*

This enzyme catalyzes the conversion of *L*-aspartate to β-alanine. Like the G3pP, this enzyme is characterized by a single substrate Michaelis-Menten kinetic with a K_m of 0.151 mM accordingly to

Ramjee et al. (1997) [52]. The same mechanism was described by Williamson and Brown (1979) [53], but with a similar K_m value of 0.16 mM, which resulted in a mean value of 0.156 mM (Table 5).

1.1.9 *L-glutamate dehydrogenase (GluD)*

The reaction catalyzed by GluD was included because the dynamic model used did not include *L*-glutamate, which is required by AspAT. GluD is responsible for the reversible conversion of the *α*-ketoglutarate to *L*-glutamate, coupled with the oxidation of NADPH to $NADP^+$. Because there were no V_{max} values concerning the direction of *α*-ketoglutarate formation, and the method used to determine the V_{max} did not allow the determination of that value, it was not possible to define the reverse reaction. The used kinetic parameters for this enzyme were found in four different works. Considering the K_m value for *α*-ketoglutarate ($K_{m,a}$) and $NADP^+$ ($K_{m,b}$), the reported values were, respectively, 0.68 mM and 0.0597 mM according to Sharkey and Engel (2008) [43], 0.64 mM and 0.040 mM by Sakamoto et al. (1975) [44], 0.46 mM and 0.012 mM by Mäntsälä and Zalkin (1976) [45], and finally, 0.2 mM and 0.035 mM by Di Fraia et al. (2000) [46]. In addition, the kinetic mechanism assumed was a simple Michaelis-Menten kinetics. These parameters were used to calculate the mean values that are reported in Table 5.

1.2 Pathways for Acrylic Acid Production

Three different heterologous pathways were identified to produce AA, the glycerol, malonyl-CoA, and *β*-alanine routes. These pathways differ from each in the production of 3-hydroxypropionate (3-HP). The glycerol pathway includes two reactions to produce 3-HP, which are catalyzed by the glycerol dehydratase (GlyDH) and the 3-hydroxypropionaldehyde dehydrogenase (3hpaD). Regarding malonyl-CoA pathway, two reactions are needed to produce 3-HP, namely the ones catalyzed by the malonyl-CoA reductase (McoaR) and the malonic semialdehyde reductase (MsaR). And finally, two for the *β*-alanine pathway, *β*-alanine aminotransferase (BaAT) and the MsaR are needed to convert *β*-

alanine to 3-HP. After obtaining 3-HP, three more enzymes are required, the 3-hydroxypropionyl-CoA synthase (3hpcoaS), 3-hydroxypropionyl-CoA dehydratase (3hpcoaDH), and the acrylyl-CoA thioesterase (AcoaTioE).

1.2.1 *Glycerol dehydratase (GlyDH)*

This enzyme is responsible for the conversion of glycerol to 3-hydroxypropionaldehyde (3-HPA). The kinetic parameters of the enzyme in *Lactobacillus collinoides* were determined by Sauvageot et al. (2002) [56]. In their work they reported a K_m of 8.3 mM for glycerol, and an activation constant (K_a) of 0.008 mM for vitamin B_{12}. Moreover, they also reported a specific activity of 0.018 $mmol.min^{-1}.mg^{-1}$ when glycerol was the substrate, and a molecular weight of 207 kDa, which allowed the calculation of a K_{cat} value of 0.0621 s^{-1}. In addition, a K_m value for glycerol (4.0 mM) reported by Schütz and Radler (1984) [57] in *Lactobacillus brevis* was also considered for the mean calculation because of the taxonomy proximity between both species. Thus, considering the available kinetic parameters, and the fact that vitamin B_{12} is an activator of the reaction and not an intermediary, the specific activation mechanism rate law was assumed (Table 6).

1.2.2 *3-hydroxypropionaldehyde dehydrogenase (3hpaD)*

The following step of the heterologous pathway is catalyzed by the 3hpaD, and it consists on the conversion of 3-HPA to 3-hydroxypropionate (3-HP) coupled with the reduction of NAD^+ to NADH. The kinetic properties of the 3hpaD from *E. coli* were studied by Jo et al. (2008) [58]. This study reported a K_{cat} of 28.54 s^{-1} when 3-HPA was used as a substrate, as well as K_m values of 0.49 mM and 0.06 mM for 3-HPA ($K_{m,a}$) and NAD^+ ($K_{m,b}$), respectively. In addition, the authors also reported product inhibition by 3-HP, with $K_{i,p}$ of 0.12 mM Considering that no kinetic mechanism was associated with this enzyme, a simple two substrate Michalis-Menten equation with product inhibition was assumed

(Table 6). Furthermore, since this reaction had no flux in the stoichiometric model when using Method 1, the V_{max} was calculated using the method for the heterologous reactions (Method 2).

1.2.3 *Malonyl-CoA reductase (McoaR)*

The McoaR is responsible for the first reaction on the malonyl-CoA pathway, which consists in the formation of malonic semialdehyde (MSA) from malonyl-CoA, coupled with the oxidation of NADPH to $NADP^+$ and the release of a coenzyme A molecule. Even though this is a reversible reaction, only the direction towards MSA formation was considered due to the lack of kinetic data in the reverse direction. This enzyme is present in *Chloroflexus aurantiacus* and was described by Hügler et al. (2002) [59], which reported a K_{cat} of 50 s^{-1} when malonyl-CoA was used as a substrate, and K_m values of 0.3 mM and 0.025 mM for malonyl-CoA ($K_{m,a}$) and NADPH ($K_{m,b}$), respectively. The authors also stated that the enzyme exhibited a two substrate Michaelis-Menten behavior (Table 6). Furthermore, the values reported by Liu et al. (2013) [60] were also accounted to determine the mean values of each parameter. They reported two K_m values for NADPH of 0.0238 and 0.0417 mM.

1.2.4 *Malonic semialdehyde reductase (MsaR)*

The MsaR enzyme, which catalyzes the final reaction of the malonyl-CoA pathway, was described by Kockelkorn and Fuchs (2009) [61]. This enzyme is responsible for the conversion of MSA to 3-HP, coupled with the oxidation of NADPH to $NADP^+$. The authors characterized this enzyme from *Metallosphaera sedula* and were able to identify its kinetic parameters. The K_{cat} value reported when malonyl-CoA was used as the substrate was 115 s^{-1}, and the K_m values were 0.07 mM for both MSA ($K_{m,a}$) and NADPH ($K_{m,b}$). Finally, a two substrate Michaelis-Menten mechanism was assumed for this model (Table 6).

1.2.5 *β-alanine aminotransferase (BaAT)*

This enzyme catalyzes the reversible conversion of β-alanine and α-ketoglutarate to MSA and *L*-glutamate. However, due to the lack of kinetic parameters concerning the reverse reaction, only the direction towards MSA formation was considered in this study. Furthermore, *E. coli*'s BaAT was described by Liu et al. (2005) [62]. The authors believed that the enzyme followed Ping-Pong Bi Bi mechanism with competitive substrate inhibition for α-ketoglutarate and proceeded to estimate the respective parameters. They reported a K_{cat} of 47.4 s^{-1}, and a K_m for β-alanine ($K_{m,a}$) and α-ketoglutarate ($K_{m,b}$), of 5.8 mM and 1.07 mM, respectively. Furthermore, the $K_{i,b}$ for α-ketoglutarate was also estimated, and was equal to 10.2 mM (Table 6). Even though this enzyme was characterized from *E. coli*, it was not present in the stoichiometric model, thus the V_{max} was calculated using the Method 2.

1.2.6 *3-hydroxypropionyl-CoA synthase (3hpcoaS)*

This enzyme uses the dephosphorylation of ATP to join a coenzyme A molecule to 3-HP, thus forming 3-hydroxypropionyl-CoA (3-HP-CoA). Alber and Fuchs (2002) [63] studied a variant of the 3hpcoaS from *Chloroflexus aurantiacus*. In their work they stated that the enzyme probably follows a Michaelis-Menten mechanism, and the parameters for that mechanism were a K_{cat} of 36 s^{-1}, and the K_m values for 3-HP ($K_{m,a}$) of 0.015 mM, CoA ($K_{m,b}$) of 0.01 mM, and ATP ($K_{m,c}$) of 0.05 mM (Table 6).

1.2.7 *3-hydroxypropionyl-CoA dehydratase (3hpcoaDH)*

The following reaction, which is catalyzed by the 3hpcoaDH, is responsible for the formation of acrylyl-CoA (AA-CoA) from 3-HP-CoA. A variant of the 3hpcoaDH from *M. sedula* was characterized by Teufel et al. (2009) [64], whose work helped to understand the underlying mechanism that controls the enzyme's activity. According to this study, the enzyme follows a Michaelis-Menten kinetics, with a K_{cat} of 96 s^{-1}, and K_m value of 0.06 mM for 3-HP-CoA (Table 6).

1.2.8 *Acrylyl-CoA thioesterase (AcoaTioE)*

The final reaction consists in the conversion of AA-CoA to AA coupled with the release of a coenzyme A molecule. No studies characterizing this enzyme's kinetic parameters were found in the literature. Hence, an enzyme catalyzing a similar reaction was sought. Ultimately, *E. coli*'s acyl-CoA thioesterase was selected as surrogate. This enzyme was the one used in the work of Chu et al. (2015) [1], which using a direct bio-based route achieved AA production for the first time. As opposed to the original reaction, this one was characterized in a report by Zhuang et al. (2008) [65], where it was reported that this enzyme kinetics follows the Michaelis-Menten model, with a K_{cat} of 0.55 s^{-1}, and K_m for AA-CoA of 0.167 mM (Table 6).

1.3 Transport Reactions

The exchange reactions allow the transport of 3-HP (XCH_3HP), AA (XCH_AA), glycerol (XCH_GLY), malonyl-CoA (XCH_MCOA), and β-alanine (XCH_BA) from the cytoplasm to the extracellular compartment and vice-versa. Since no kinetic data concerning transport reactions for these metabolites was found or even evidence of the existence of such mechanisms in the case of 3-HP and AA, these reactions were included akin to the ones in the original model [18]. Furthermore, this entire set of reactions follows the reversible Michaelis-Menten kinetics, with a V_{max} of 100 mM/s and a K_m of 10 mM, as it was arbitrarily chosen in the original model [21].
