# Supplementary material for: A kinetic model of the central carbon metabolism for acrylic acid production in Escherichia coli
Source: PLoS Comput Biol. 2021 Mar 8;17(3):e1008704. doi: 10.1371/journal.pcbi.1008704 (PMC7971886; doi:10.1371/journal.pcbi.1008704)
Supplement: Attachment — Submitted filename: Revision_Response.pdf [file pcbi.1008704.s015.pdf]

A kinetic model of the central carbon metabolism for acrylic acid production in *Escherichia coli*

Alexandre Oliveira, Joana Rodrigues, Eugénio Ferreira, Lígia Rodrigues, Oscar Dias

Reviewer #1

First Comment:

"The kinetic model used in this study was initially developed, validated and analyzed for E. coli grown on glucose, which is one of the condition analysed in this study. The model is thus expected to be valid in this condition. However, the authors also investigate production on glycerol, which enters into glycolysis at the level of trioses phosphate and results in a significant change of intracellular fluxes. How close is the flux distribution predicted by the model on glycerol (in absence of production) compared to the flux distribution measured on this carbon source (e.g. obtained by 13 C-metabolic flux analysis, DOI 10.1016/j.jbiosc.2017.09.009)? This should be evaluated and briefly discussed (either in the main text – at least mentioned here – or in supplementary data). This would strengthen the predictions obtained on glycerol, and could be used to validate some methodological aspects (e.g. to evaluate the relevance of the method used to estimate unknown parameters)."

Response:

We appreciate the reviewer's comment. Indeed, the flux distribution is different from the distribution measured experimentally in the mentioned study [1] and other studies [2]. The main difference is that the model does not direct as much carbon towards the synthesis of glucose-6-phosphate, and consequentially the flux towards the pentose-phosphate pathway is much lower. Conversely, the flux towards the TCA cycle is much higher. Hence, a statement (**line 103-109**) and a new section in S1 Appendix (**line 2-23**), raising awareness to this fact, was included in the main manuscript:

"Although the original model was developed and validated for growth on glucose, the steady-state flux distribution (when using glycerol as carbon source) was compared with values determined experimentally [12,13]. This assessment unveiled a significantly

different flux distribution between the dynamic model and experimental data (S1 Appendix, section 1.1), which was considered when analysing the results under glycerol consumption. Addressing these differences would require determining the parameters for most reactions, which was not the goal of this work. Nevertheless, it would be a relevant topic to address in future work in order to improve the quality of the models."

Second Comment:

Part 1 - "Most reactions of the production pathways are modelled as an irreversible process. However, there are many examples where product accumulation inhibits the flux, due for instance to thermodynamics constraints, and this is not taken into account in the model. For instance, the production of acetate from glucose decreases as soon as acetate concentration reaches a few millimolar (DOI 10.1038/srep42135). Consistently, in some situations, the end-products must be removed from the environment of cells to maintain a production (e.g. DOI 10.1016/s0167-7799(99)01351-7). I am not sure whether this would affect the outcome of this work or not, but I think it would be worth implementing reversible rate laws (several generic reversible rate laws are available in the literature)."

Response:

We acknowledge the reviewer for mentioning this fact, with which we agree. In fact, when collecting the kinetic parameters for each reaction, we first focused on determining their reversibility. However, to the best of our knowledge, most reactions defined as reversible in databases, like BRENDA and BioCyc, lack kinetic information on the reverse direction. Actually, in some cases, it was also not possible to account for product inhibition due to the lack of inhibition constants.

Hence, although we agree with the reviewer, it was not possible to include such information in the model.

Part 2 - "Similar considerations about the glycerol uptake pathways could also explain why some models were "unable to reach a steady-state" (lines 194, 204, 229) in some conditions, and why "model presented issues with dihydroxyacetone accumulation" (line 191)."

Response:

We agree with the reviewer that these considerations could influence the results obtained when seeking a steady-state. Furthermore, we acknowledge that the GlyD reaction should also be reversible; however, as the rate law required a Vmax for the reverse reaction, and we could not determine such parameter with these methods, we could not account for the reversibility of the reaction. This information was disclosed **in lines 44-47** of **S2 Appendix, section 1.1.4**.

Part 3 - "At the level of the production pathway, the malonyl-CoA reductase catalyzes the reaction McoaR, which is defined as reversible in Brenda and other databases. Similarly, the reaction catalysed by the beta alanine aminotransferase (BaAT) is reversible (most reactions catalyzed by aminotransferases are freely reversible, with ΔG°' ≈ 0 kJ/mol)."

Response:

Again, we agree with the reviewer regarding the reversibility of the reactions. We tried to set reactions as reversible when there was evidence that they are reversible. However, the availability of parameters was once more a limiting factor. For instance, the McoaR reaction is indeed defined as reversible in BRENDA and other databases. Yet, we only found kinetic parameters for the direction of malonic semialdehyde formation. Since reversible rate laws must include the maximal rate in the reversible reaction and the Km of the products, it was not possible to incorporate the kinetics of the reversible reaction. The same for the BaAT, while it is well established that the reaction is reversible, the only characterization of the enzyme's kinetics in *E. coli* that was found just includes the direction of malonic semialdehyde formation. Thus, the reversible reaction could not be included. A statement was included in the **supplementary material 2, lines 135-137**, for the McoaR, and **line 152-154**, for the BaAT, to clarify these situations.

Part 4 - "3-HPA dehydrogenase is also subject to product inhibition (e.g. see DOI 10.1038/s41598-017-15400-x). Please check for other reactions/enzymes, and modify the model accordingly."

Response:

We acknowledge the reviewer for this suggestion, with which we agree. As for the product inhibition in the 3-HPA dehydrogenase, the mentioned article did not present an inhibition constant for 3-HP. However, after re-reading the paper that provided the kinetic parameters used for developing these models [3], they do mention a Km value for 3-HP of 0.12 mM, which can be used to define the substrate inhibition. Although the models were updated accordingly, the final concentration of AA using both substrates did not change. Hence, the rate law and the parameters for this enzyme were updated in **table 5**, and the inhibition constant was added in the **supplementary material 2 lines 127-129**.

Third Comment:

Part 1 - "Most of the Vmax values used in the model are in a reasonable range, but some values are far too high. For instance, the Vmax of AccC is set to 0.568 mM/s (i.e. mmol/L_cytosol/second), which corresponds to 3.6 mmol/gDW/h and is in a correct range. In contrast, the Vmax of AspAT is set to 127 mM/s, which corresponds to 809 mmol/gDW/h and is much above a biologically plausible value. This might indeed be "ideal for maximizing the production yields" (lines 236-237) but is hardly conceivable in practice. Some of the tested Vmax values are even higher (e.g. for some simulations the Vmax of GlyD was set to 4300 mM/s, which corresponds to 27400 mmol/gDW/h!!, S1 appendix)."

Response:

We appreciate the reviewers concerns and acknowledge the opportunity to clarify their doubts. Regarding the AspAT, the original Vmax is 2.5548 mM/s and the value of 127 mM/s is the result of the *in silico* optimization. As stated in the manuscript, the goal of the optimization was to identify potential targets to serve as a guideline for future *in vivo* experiments, and not to predict the production of AA accurately. Even though 127 mM/s is considerably above a biologically plausible value, here the goal was to identify the AspAT reaction as a potential target for optimization. To make that clearer the below statement was added to **lines 280-282:**

"It is worth noting that, even though this value is significantly higher than what is biologically feasible, the goal of the optimisation was to identify potential targets and not meticulously predict the final V_{max} value.".

Part 2 - "Parameters values should be kept within a biologically feasible range. The constraints should thus be adapted accordingly during model construction and optimization. This might be related to the enzyme concentration assumed for all reactions, which was 100 mM. This corresponds to roughly 10^8 copies of proteins per E. coli cell, i.e. much higher than the concentration of the most abundant enzymes. This value is two orders of magnitude higher than the protein expression limit estimated in yeast (DOI 10.1091/mbc.E15-07-0512) and about two orders of magnitude higher than the entire proteome of an E. coli cell (estimated at 3.10^6 copies per cell, bionumbers ID 100088)."

Response:

We do agree with the reviewer concerning the excessive enzyme concentration used in this work for the heterologous reactions. However, that was the purpose of selecting such value. Since no Vmax value was found, it was calculated by multiplying the turnover (Kcat) by the enzyme concentration. Moreover, as we also could not find absolute quantification data for these enzymes, we defined an excess concentration of 100 mM to calculate the Vmax, as previously used in similar works [4]. Even though that value is significantly higher than what is biologically plausible, selecting a lower value could create artificial bottlenecks in the heterologous pathway that would impair the search for potential optimization targets in the central carbon metabolism (CCM).

Nevertheless, as suggested by the reviewer, we tested different enzyme concentrations ([Etot] = 10 and 1 mM) to assess the model's sensitivity to this parameter.

A 10 mM concentration had no influence in the simulations and there was no bottleneck created. Regarding, the 1 mM concentration the results of the Gly_Gly model were severely impaired. These results were added as a new section (**section 1.5**) to the **S1 Appendix** and mentioned in a paragraph (**line 350-353**):

"Even though an enzyme concentration of 100 mM is beyond what is biologically feasible, this value was selected to avoid creating artificial bottlenecks that would impair this analysis. Furthermore, the authors also tested different concentrations to assess the impact on AA production and the results are presented in S1 Appendix, section 1.5."

However, the value was kept at 100 mM since this value was already established in the literature [4].

Fourth Comment:

Part 1 - "For batch simulations, the substrate (glucose or glycerol) is consumed at a constant rate, and the product accumulates at a constant rate. A similar behaviour is observed for all simulated profiles. This should not be the case in batch condition (due to exponential growth of E. coli). These artifactual simulated profiles emerge from defining biomass concentration as constant. When having a look at the models, we can see that growth is indeed simulated, but there is no accumulation of biomass (which is not even defined as a species). Hence, the authors cannot state that "a batch version of the model was used for the optimisation" (line 395 and elsewhere)."

Response:

We acknowledge the reviewer for pointing this out, which allows us to clarify our claims. When we stated that a "batch version of the model was used", we were highlighting that, on the one hand, we used a continuous model (with a feed and drains), to determine the FCC values, and on the other hand a model simulating batch conditions with only an initial concentration of glucose and no drains was used for optimizations.

Nevertheless, we agree with the reviewer that it cannot be stated that these are batch models. Hence, we have rewritten **lines 442-444** on the manuscript to disclose that more clearly:

"The optimisation was then performed, using the automated optimisation tool provided by COPASI [28], for the initial models with only an initial concentration of glucose (10 g/L) and without drains for the end metabolites."

Moreover, the original model did not account for biomass accumulation as the authors assumed that it followed an exponential growth rate in a continuous system [5]; thus, remaining constant. Nevertheless, the model includes a pseudo-reaction that simulates the growth of the microorganism, though not affecting the cellular volume, which is often the case with kinetic models [4–10].

Part 2 - "This should be corrected (by defining biomass as a specie and taking into account its concentration in the changes of extracellular species – substrates and products -)."

Response:

To the best of our knowledge, we found very few relevant models that simulate a batch culture with biomass accumulation [11–14]. We focused on the Jahan model [11] to use as a reference to define biomass as a species. These models determined the growth rate as a function of ATP production, and then used it to simulate biomass concentration, which in turn affect uptake and excretion rates.

We tried to replicate that using the flux of the Growth reaction of the Millard model [5], as the growth rate for a biomass species. We found that the final AA concentration would be directly impaired by the initial biomass concentration. Therefore, we tested different values to determine their impact. Initially, we used the same value used for the Jahan model (0.012 mM) [11]. However, such a low value means a lower rate of transport of glucose into the cytosol, which impacted AA production. Ultimately, the growth stopped early, causing the saturation of the glucose transport, and thus most provided carbon was not consumed. Next, we tried to determine the initial biomass concentration by performing a steady-state analysis, as this strategy was used by Chassagnole et al [6] to determine the initial conditions for the dynamic model. Therefore, we used the extended CCM models to perform a steady-state analysis by default, and the resulting concentration was 0.1139 mM. When performing a time course simulation using that concentration, as for the previous concentration, the models were not able to consume most of the provided carbon.

Finally, we tried to determine the biomass concentration by converting the biomass concentration from gDW/L to mmol/L. First, we gathered results of the initial OD600 measurements, from reports in which 3-HP and AA production was tested *in vivo* (the most frequent value was 0.6). Then, we searched the Bionumbers database for conversion factors that would allow us to obtain the gDW/L concentration (ID: 109835 [15], 109837 [16], 109838 [17] – average value of 0.33 gDW/L for one unit of OD600). Finally, using the elemental composition of *E. coli* (Bionumbers ID: 106666 [18]), and their respective molecular weights, we were able to obtain a cellular concentration of 2.47 mmol/L.

With this concentration the model was able to consume all glucose provided; though, at a slower rate (from three hours to eight hours). This also affected AA production, as the final concentration almost doubled in the Glc_Gly and Glc_Mcoa model, and was about six time higher in the Glc_Ba model. Nevertheless, the Glc_Mcoa model also presented some issues as the glucose consumption stopped at 10mM. Concerning the models that use glycerol as carbon source, as the model was not originally designed to use this carbon source, the internal fluxes changed drastically, as already discussed in the first comment of the reviewer. These changes affected the flux of the growth reaction, which quickly becomes zero after the start of the simulation.

These results showed that although desirable, more experimental data would be required to realistically incorporate the biomass accumulation in our model.

Fifth Comment:

"There is no validation of a major outcome of this study, i.e. the identification of reactions that control AA production. This is currently lacking and would greatly improve the manuscript. In my opinion, it is important to validate (at least qualitatively) that increasing the rates of the proposed controlling reactions actually translates into an increased production of 3-HP or AA. This could be done by quantifying production in strains overexpressing (for instance on a plasmid) enzymes that catalyse the identified reactions."

<u>Response:</u>

We acknowledge the importance of experimental validation in this kind of studies. Unfortunately, this work was designed to focus only on the computational part, and it was not possible to include any *in vivo* study. We would like to implement these pathways *in vivo* in the future and validate the results presented in this manuscript, but that is currently out of scope. Nevertheless, like in other works published without experimental validation to support their findings [19,20], we think that these findings are relevant and may help to improve and accelerate other studies.

Furthermore, we also supported the targets proposed on the optimization task with experimental works found in the literature, namely:

1. <u>***Regarding the G3pD reaction -*** *"To the best of our knowledge, there are no evidences in literature regarding the use of this reaction as a target for*</u>

optimisation, as this pathway is mainly used to produce 3-HP or AA from glycerol. Moreover, only Chu et al. (2015) [1] used glucose for AA production; however, they focused their optimisation strategies on limiting the toxicity of intermediary compounds." (**Lines 235-239**)

2. ***Regarding the AccC reaction*** - *"Moreover, this is a well-established target for optimisation of the malonyl-CoA pathway [19]."* (**Lines 264-265**)
3. ***Regarding the AspC reaction*** - *"This reaction allows converting oxaloacetate and L-glutamate into aspartate, which is in turn converted to β-alanine. Moreover, reports from the literature suggest that increasing the bioavailability of aspartate leads to a higher 3-HP production, as in vivo experiments with Saccharomyces cerevisiae and E. coli showed it as viable optimisation strategy [11,21]."* (**Lines 274-276**)

Sixth Comment:

"All models are available at https://cutt.ly/aaKineticModels, thanks for sharing these models with the community. However, this address appears to refer to an URL shortener service, which cannot guarantee their long-term availability. Please, provide models via dedicated repositories (such as biomodels database) to ensure long-term availability."

Response:

We agree with the suggestion and have currently submitted the model to the Biomodels Database and the Biomodels identifiers of each model were added to manuscript and S1 Appendix **(S1 Table 1),** and referenced in the main manuscript (**lines 93-94; lines 422-423**). However, the models will only be available publicly when the manuscript is published. Hence, we were not able to include the links to the Biomodels database. To circumvent that we will keep the previous link also active (https://cutt.ly/aaKineticModels) so that the reviewers can still have access to the models.

First Minor Comment

"The models and the general approach used in this work are not described at all in the results section (though all relevant information is given in the methods

section and in supplementary information). It would be worth adding a paragraph at the beginning of the results to briefly describe the models (structure, construction approach, limitations…) and refer to the methods section for more detailed information. Lines 277-280 could be moved to this paragraph. This would make the manuscript easier to read."

Response:

We agree with the reviewer comment and added two new sections (**lines 89-102 and lines 110-116**) to the main manuscript, and a section in the **S1 Appendix (Line 24-29)** that summarize the details of all the models that resulted from this work:

"**Extended Central Carbon Metabolism (CCM_extended) model**

Initially the original model of the CCM was extended to include the production of glycerol, malonyl-CoA, and β-alanine, from glucose (CCM extended Glc), resulting in a model with 87 reactions and 88 metabolites. This model is available at the Biomodels database with the identifier MODEL2010030001. Additionally, two more reactions were added to the CCM, which resulted in a new model with 89 reactions and 89 metabolites (Biomodels ID: MODEL2010160002), as the added reactions did not allow using glycerol as a carbon source. However, the latter model was not used to simulate the production of 3-HP and AA from glucose, as the it was not possible to determine all parameters of the GlyD reaction, which is responsible for the reversible conversion of glycerol to dihydroxyacetone. Method 1 only allowed the estimation of the Vmax parameter in the direction of dihydroxyacetone formation, due to the limitations of the stoichiometric model's flux balance analysis. Hence, only this direction was considered for the model, thus affecting the dynamic model behaviour when using glucose as a carbon source, as instead of contributing for glycerol biosynthesis, the reaction would deflect glycerol towards the CCM."

"3-Hydroxypropionate and Acrylic Acid Producing Models

The CCM extended model was then used as a chassis, in which the three heterologous reactions were separately integrated, to simulate in silico production of 3-HP and AA from both carbon sources and to determine which is associated with higher yields. Twelve different dynamic models were generated, and the details of each model are presented in S1 Appendix, section 1.2. Moreover, during simulations, several issues

arose, leading to variations in parameters before the analysis of the 3-HP and AA production. These variations are explained in detail in the S1 Appendix, section 1.3."

Second Minor Comment

"Please check the figures (and tables) numbering in the text (I think most of them are wrong, e.g. Fig 2 in the text line 313 should not refer to simulation results but to the production pathways, which are shown in Fig 1)"

Response:

We acknowledge the reviewer for this correction for which we apologize. It seems there was an auto numbering issue in the original submission. We addressed this issue by correcting the figure numbering in **S1 and S2 appendices**, and the following lines of main manuscript: **120, 122, 124, 138, 163, 164, 166, 181, 182, 186, 211, 233, 243, 264, 268, 273, 283, 291, 355, 360, 365, 384, 385, 416.**

Third Minor Comment

"Line 139: "the behaviour analysis of the model…" Which model? Glu-Mcoa or Gly-Mcoa?"

Response:

We acknowledge the reviewer for pointing out this unclear phrase. The model is the Gly-Mcoa, thus we changed the sentence in **lines 164-166** to *"The behaviour analysis of the Gly-Mcoa model showed a considerable intracellular accumulation of dihydroxyacetone (Fig 4C)."*

Fourth Minor Comment

"Line 184: Reference to Table 5 should be Table 3. The results obtained for the beta-alanine pathway are not so "promising" (line 183) since this pathway has by far the lowest predicted and measured titer (as shown in table 3)"

Response:

We accepted the correction concerning the change of table 5 to table 3. However, we did not intend to say that the beta-alanine pathway was promising, instead that the model had promising predictions since the AA concentration was close to what

was produced *in vivo* by Song et al. (2016) [21]. To clarify the message for readers, we updated the statement in **line 209-211** to *"Nonetheless, the β-alanine model predictions showed promising 3-HP yields, as the projected concentration was close to the results obtained in vivo by Song et al. (2016) [11] (Table 3)."*

Fifth Minor Comment

"Line 261: "with the useD of glycerol" => "with the use of glycerol""

Response:

We acknowledge the reviewer for this correction, which we accepted included in the revised manuscript.

Sixth Minor Comment

"GluD is defined as a reversible reaction (equation 9), but the rate law corresponds to an irreversible reaction (table 5). Please correct."

Response:

We agree with the reviewer that the GluD is a reversible reaction, and that there was a contradiction between equation 9 and the reversible rate law presented in table 5. However, the GluD reaction is another case in which the reaction was reversible but it was not possible to account for reverse reaction due to the lack of kinetic data. In this case, even though we found Km values for the products, a Michaelis-Menten Reversible rate law require a Vmax for the reaction in the opposite direction, which was not possible to determine with the method we used to determine the Vmax for this reaction. Hence, we included the reaction as an irreversible reaction.

Therefore, we updated **equation 9**, and included a statement in the supplementary material to disclose that even though the reaction was reversible, it was not possible to include the reverse reaction due to lack of data (**supplementary material 2 lines 89-92**). Furthermore, when re-analysing this reaction, we found a value of a parameter (Km for NADPH) that was incorrect. Therefore, we updated the Km value (**Table 5**) and the results for the Beta-alanine pathway in **lines 187-189**, **table 3**, **figure 6** and **figure 7,** although the new results still support the original conclusions.

Seventh Minor Comment

"Equation (13) which describes the 3-hydroxypropionaldehyde dehydrogenase (3hpaD) reaction: the product is 3-HP, not 3-HPA. Please correct."

Response:

The reviewer is correct; therefore, the product in **equation (13)** was changed from 3-HPA to 3-HP.

Reviewer #3

First Comment:

"The methods for determining initial conditions of the models are especially opaque – I can hardly find any details at all of how the initial steady state of the model was determined. This has direct implications to whether the agreement between data and model titers in Table 2 was fit by the authors or emergent from model behavior. The latter is obviously more impressive, but fitting to expected behavior is fine as well, as long as some type of model validation is presented elsewhere."

Response:

We acknowledge the reviewer concerns, comments and suggestions and the opportunity to improve the manuscript and make it clearer. The initial steady-state was determined using the default model. No changes were made to the default settings of the steady-state task in COPASI. We used the Integration and Newton methods, with a resolution of $1x10^{-9}$, derivation factor of 0.001, iteration limit of 50 steps and a target criterion of distance and rate. Moreover, no further fitting to the expected behaviour was performed. We added this information to the manuscript in **lines 333-335** to clarify this methodology:

"Initially, a steady-state flux distribution is determined for the original kinetic model, using the default settings of the COPASI software [28] steady-state task and not further fitted to the expected behaviour."

Second Comment:

"Method 1 for parameterization, which uses an estimate of the kinetic v_max calculated by Flux Variability Analysis, is problematic inherently. The v_max term in FVA and the Michaelis-Menten kinetic v_max term are completely different fundamentally. The former v_max is a theoretical value having to do with the constraints on the system's mass balances given measured metabolic exchanges and a growth rate. For many reactions, v_max solved with FVA may even hit 'infinity' due to loops in the network, or may be impractically high because of a lack of known constraints on the pathway. Although they share a name, the FVA v_max is very different then the kinetic v_max, which is simply the maximum rate that a certain mass

of enzyme can catalyze a reaction, approximated by E_tot*k_cat. To justify the use of Method 1 for estimating the latter v_max parameters, the authors need to perform validation by reproducing measured kinetic v_max values with their method, i.e. extend their S1 Table 2 calculations to reactions with measured v_max and compare. My suspicion is that the authors would be better off inserting a physiological flux value rather than an FVA-calculated maximum flux value, for example as has been done in previous work for estimating enzyme turnover rates (see PMID: 26951675)."

Response:

We acknowledge the reviewer's definitions; with which we agree. However, we do not claim that both terms have the same meaning. Actually, the difference in the terms' definitions is what we want to explore for bounding the kinetic Vmax. We assume that the stoichiometric model operates in ideal conditions, and it is assumed that the prediction of reaction's maximum flux can be used as an excessive approximation to the enzyme's Vmax. Moreover, the stoichiometric model's simulations did not hit infinite loops, as most *E. coli* models are well curated and do not have this issue.

This is a well-established methodology to determine Vmax values, as it was first used by Chassagnole et al. (2002) [6]. This strategy has been successfully used in subsequent works, to estimate kinetic Vmax values when *in vivo* experiments are not available. Furthermore, as stated by reviewer 1, this method is capable of providing reasonable results that are in the range of biologically plausible values. Regarding the suggestion to estimate the physiological flux value, although being desirable, we could not find data for the enzymes required to extend the central carbon metabolism.

Third Comment:

"I also do not understand the authors' justification for Method 2. They state that in cases where the heterologous enzyme is high, then v_max = k_cat * E_tot. In a simple Michaelis-Menten derivation, then this equation is the definition of the v_max term, so I don't know why the enzyme concentration is relevant. If the authors are saying the v = v_max at high enzyme levels, then this seems problematic from the factor that at very high enzyme concentrations (100mM as they state), the substrate is not likely to be high enough concentration to saturate enzyme sites, so v would be

much less than v_max. Hopefully the authors can clarify better how Method 2 works and better justify it as necessary.”

Response:

We are pleased to clarify the reviewer’s doubts. Regarding the heterologous pathways, two issues arose when collecting kinetic data.

1. The first one concerned the native reactions of *E. coli*. The Vmax values are rarely reported when assessing the kinetic properties of enzymes, usually turnover and specific activity values are reported instead.
2. The second was that for heterologous reactions, and some reactions present in *E. coli*, it was not possible to use method 1 to determine a Vmax value.

Therefore, we relied on the definition to determine the Vmax, as the enzyme turnover (Kcat) values were available, and only an enzyme concentration was required. However, like before for the Vmax parameter, such concentration is seldom reported when kinetic characterizations are published. Hence, our strategy was to assume that such reactions were not bottlenecks, which would impair this analysis as the objective is studying how the central carbon metabolism affects AA production, assuming excess enzyme. This strategy was already applied in the work of Machado et al. [4]

Moreover, we sought proteomics data to find absolute enzyme quantification, but again to the best of our knowledge, the lack of data impaired the search.

Fourth Comment:

“In general, it would be nice if the model predictions were presented more systematically in some format. The existing tables are quite nice, but the models’ many predictions and failure modes are discussed haphazardly throughout the manuscript which makes it a bit difficult to summarize the designs of greatest excitement. Tables 2 and 4 do a good job, but even more summary like this would be nice if possible.”

Response:

We appreciate and agree with the reviewer comments. Indeed, we chose to use the conclusion section to summarize the findings of this study and the issues the models presented during the simulations.

Therefore, to address this, we included a paragraph at the end of the time course simulations section (**lines 215-224**) to discuss which pathways seem more profitable for industrial-scale production:

"When comparing the three bio-based routes, the results suggest, as supported by literature [1,19], that the glycerol pathway leads to the highest yields, when combined with the use of glycerol as a carbon source. However, a relevant caveat must be recalled. This pathway includes a reaction that relies on the presence of vitamin B12, which represents a significant economic disadvantage at an industrial-scale production [8,9]. Hence, to make this route economically viable, either the yield must be significantly improved to overcome the cost of the vitamin supplementation, or a cheaper path to produce B12 must be found. Therefore, despite producing less AA, it seems to be beneficial to use the malonyl-CoA route, as it provided the second-highest yield and does not require vitamin supplementation [18,19,21]. Nevertheless, the pathway should still be optimised to obtain yields that could compete with the existing methods at an industrial-scale production."

Moreover, we also added a statement at the end of discussion of the optimization section (**line 294-297**):

"It is important to notice that the four targets suggested by this analysis aim at increasing the bioavailability of the intermediaries (glycerol, malonyl-CoA, or β-alanine). Nevertheless, these models only comprise the CCM. Thus, other strategies to force additional flux towards the heterologous pathway may also prove useful."

Furthermore, we also supported the results of the optimization task with experimental works that were found in the literature:

1. ***Regarding the G3pD reaction -*** *"To the best of our knowledge, there are no evidences in literature regarding the use of this reaction as a target for optimisation, as this pathway is mainly used to produce 3-HP or AA from glycerol. Moreover, only Chu et al. (2015) [1] used glucose for AA production; however, they focused their optimisation strategies on limiting the toxicity of intermediary compounds."* (**Lines 235-239**)

2. ***Regarding the AccC reaction -*** *"Moreover, this is a well-established target for optimisation of the malonyl-CoA pathway [19]."* (**Lines 264-265**)
3. ***Regarding the AspC reaction -*** *"This reaction allows converting oxaloacetate and L-glutamate into aspartate, which is in turn converted to β-alanine. Moreover, reports from the literature suggest that increasing the bioavailability of aspartate leads to a higher 3-HP production, as in vivo experiments with Saccharomyces cerevisiae and E. coli showed it as viable optimisation strategy [11,21]."* (**Lines 274-276**)

Finally, the conclusion section (**line 301-314**) was also updated to avoid redundancies with statements previously added:

"In conclusion, these models seem to be more accurate in predicting 3-HP synthesis as the Vmax for the heterologous enzymes was calculated in excess, not to limit the reaction flux. The models exhibited limitations regarding the assimilation of glycerol and β-alanine production. An effort was put forward towards finding proteomics data that included the absolute quantification of such enzymes to solve these problems. However, to the best of our knowledge, no quantification data was found; therefore, it will be important in the future to seek such data or, in the lack of new data, to determine it experimentally. Moreover, this analysis indicates that, even though not exhibiting the highest yields, the malonyl-CoA path appears to be the best choice for industrial-scale production of AA, as it does not require vitamin supplementation and there is still room for optimisation. As for the comparison between glucose and glycerol, an overall best carbon source does not emerge from this work. Instead, the answer is specific to the selected pathway. Finally, this study also suggests four optimisation targets that, theoretically, should result in higher yields. Nonetheless, as this study only focused on the computational work, validation with in vivo experiments is required in the future to further confirm these results."

Reviewer #4

First Comment:

"First, the model should be clearly presented as how many reactions and metabolites and what the constraints are. all these are lacking in the study."

Response:

We agree with the reviewer comment and added two new sections (**lines 89-102 and lines 110-116**) to the main manuscript, and a section in the **S1 Appendix (Line 24-29)** that summarize the details of all the models that resulted from this work:

"**Extended Central Carbon Metabolism (CCM_extended) model**

Initially the original model of the CCM was extended to include the production of glycerol, malonyl-CoA, and β-alanine, from glucose (CCM extended Glc), resulting in a model with 87 reactions and 88 metabolites. This model is available at the Biomodels database with the identifier MODEL2010030001. Additionally, two more reactions were added to the CCM, which resulted in a new model with 89 reactions and 89 metabolites (Biomodels ID: MODEL2010160002), as the added reactions did not allow using glycerol as a carbon source. However, the latter model was not used to simulate the production of 3-HP and AA from glucose, as the it was not possible to determine all parameters of the GlyD reaction, which is responsible for the reversible conversion of glycerol to dihydroxyacetone. Method 1 only allowed the estimation of the Vmax parameter in the direction of dihydroxyacetone formation, due to the limitations of the stoichiometric model's flux balance analysis. Hence, only this direction was considered for the model, thus affecting the dynamic model behaviour when using glucose as a carbon source, as instead of contributing for glycerol biosynthesis, the reaction would deflect glycerol towards the CCM."

"3-Hydroxypropionate and Acrylic Acid Producing Models

The CCM extended model was then used as a chassis, in which the three heterologous reactions were separately integrated, to simulate in silico production of 3-HP and AA from both carbon sources and to determine which is associated with higher yields. Twelve different dynamic models were generated, and the details of each model are presented in S1 Appendix, section 1.2. Moreover, during simulations, several issues

arose, leading to variations in parameters before the analysis of the 3-HP and AA production. These variations are explained in detail in the S1 Appendix, section 1.3."

Second Comment:

"Second, these three pathways work in a network which includes three of them operating simultaneously (I suppose or the authors should indicate otherwise), so they cannot be modeled as individual pathways as they were in the study."

Response:

We acknowledge the reviewer for this comment that allows us to make the manuscript clearer.

As stated in **lines 62-66** of the introduction, these pathways are heterologous and three different alternatives are tested to produce 3-HP. The various AA synthesis heterologous pathways, presented in this manuscript only differ from each other on the steps towards 3-HP production, and then share the same reactions that convert this intermediary into AA (**Fig. 1**). Hence, they should not be modelled as a network, as shown in the articles cited in **table 1**, **table 2**, and **table 3**, in which AA production *in vivo* was achieved using exclusively one of the three pathways.

Nevertheless, we acknowledge that the message was not clear in the first paragraph in the materials and methods. Hence, the first paragraph (**Line 317-321**) was updated to:

"The dynamic model developed by Millard et al. (2017) [22] was used as a chassis to insert the heterologous pathways. However, the model did not include the production of glycerol, malonyl-CoA and β-alanine, which are naturally produced in E. coli. Therefore, the first step was to extend the original model to include these metabolites. Subsequently, each heterologous pathway was added separately to compare 3-HP and AA synthesis."

Moreover, a section with the native central carbon extended model (CCM_extended) was added to the manuscript in **lines 89-102**. This model was used as the basis for building the heterologous models and contains the reactions required to produce the three intermediary compounds (glycerol, malonyl-CoA and β-alanine) that were not available in the original Millard model [5]. Only the three intermediaries were included simultaneously in the CCM_extended model since they all are native to *E. coli*.

Third Comment:

"Third, for this kind study, some kind of experimental validation is required."

Response:

We acknowledge the importance of experimental validation in this kind of studies. Unfortunately, this work was designed to focus only on the computational part, and it was not possible to include any *in vivo* study. We would like to implement these pathways *in vivo* in the future and validate the results presented with this manuscript, but currently this is out of scope. Nevertheless, like in other works published without experimental validation to support their findings [19,20], we think that these findings are relevant and may help to improve and accelerate other studies.

Furthermore, we also supported the results of the optimization task with experimental works that were found in the literature:

1. ***Regarding the G3pD reaction*** - *"To the best of our knowledge, there are no evidences in literature regarding the use of this reaction as a target for optimisation, as this pathway is mainly used to produce 3-HP or AA from glycerol. Moreover, only Chu et al. (2015) [1] used glucose for AA production; however, they focused their optimisation strategies on limiting the toxicity of intermediary compounds."* (**Lines 235-239**)
2. ***Regarding the AccC reaction*** - *"Moreover, this is a well-established target for optimisation of the malonyl-CoA pathway [19]."* (**Lines 264-265**)
3. ***Regarding the AspC reaction*** - *"This reaction allows converting oxaloacetate and L-glutamate into aspartate, which is in turn converted to β-alanine. Moreover, reports from the literature suggest that increasing the bioavailability of aspartate leads to a higher 3-HP production, as in vivo experiments with Saccharomyces cerevisiae and E. coli showed it as viable optimisation strategy [11,21]."* (**Lines 274-276**)

Fourth Comment:

"Fourth, Kcat for Vmax and the parameters estimated from FBA of a global model for a subnetwork are questionable, although I understand these parameters may be not available otherwise."

Response:

Both strategies were implemented to circumvent the lack of available kinetic data. Therefore, we used well-established techniques to deal with the lack of data. The strategy to estimate the Vmax from FBA is a well-established methodology as it was first used by Chassagnole et al. (2002) [6]. Furthermore, this strategy has been successfully used in subsequent works, to estimate Vmax values when *in vivo* experiments are not available. As stated by reviewer 1, this method is capable of providing reasonable results that are in the range of biologically plausible values. The other strategy was used because the only available parameter that allowed Vmax estimation was the Kcat, and only an enzyme concentration was required to determine the values. However, like for the Vmax parameter, such concentration is never reported when kinetic characterizations are published. Hence, our strategy was to assume that these reactions were not bottlenecks, which would impair this analysis as the objective is studying how the central carbon metabolism affects AA production, by assuming excess enzyme. This strategy was already applied in the work of Machado et al. [4].

References:

1. Toya Y, Ohashi S, Shimizu H. Optimal 13C-labeling of glycerol carbon source for precise flux estimation in *Escherichia coli*. J Biosci Bioeng. 2018. doi:10.1016/j.jbiosc.2017.09.009;
2. Yao R, Li J, Feng L, Zhang X, Hu H. 13 C metabolic flux analysis-guided metabolic engineering of *Escherichia coli* for improved acetol production from glycerol. Biotechnol Biofuels. 2019. doi:10.1186/s13068-019-1372-4;
3. Jo J-E, Raj S, Rathnasingh C, Selvakumar E, Jung W-C, Park S. Cloning, expression, and characterization of an aldehyde dehydrogenase from *Escherichia coli K-12* that utilizes 3-hydroxypropionaldehyde as a substrate. Appl Microbiol Biotechnol. 2008;81: 51.;

4. Machado D, Rodrigues LR, Rocha I. A kinetic model for curcumin production in *Escherichia coli*. Biosystems. 2014;125: 16–21. doi:10.1016/j.biosystems.2014.09.001;

5. Millard P, Smallbone K, Mendes P. Metabolic regulation is sufficient for global and robust coordination of glucose uptake, catabolism, energy production and growth in *Escherichia coli*. Maranas CD, editor. PLOS Comput Biol. 2017;13: e1005396. doi:10.1371/journal.pcbi.1005396;

6. Chassagnole C, Noisommit-Rizzi N, Schmid JW, Mauch K, Reuss M. Dynamic modeling of the central carbon metabolism of *Escherichia coli*. Biotechnol Bioeng. 2002;79: 53–73. doi:10.1002/bit.10288;

7. Schmid JW, Mauch K, Reuss M, Gilles ED, Kremling A. Metabolic design based on a coupled gene expression - Metabolic network model of tryptophan production in *Escherichia coli*. Metab Eng. 2004. doi:10.1016/j.ymben.2004.06.003;

8. Pontes Freitas Alberton K, Alberton AL, Di Maggio JA, Estrada VG, Díaz MS, Secchi AR. Simultaneous parameters identifiability and estimation of an *E. coli* metabolic network model. Biomed Res Int. 2015. doi:10.1155/2015/454765;

9. Peskov K, Mogilevskaya E, Demin O. Kinetic modelling of central carbon metabolism in *Escherichia coli*. FEBS Journal. 2012. doi:10.1111/j.1742-4658.2012.08719.x;

10. Khodayari A, Zomorrodi AR, Liao JC, Maranas CD. A kinetic model of *Escherichia coli* core metabolism satisfying multiple sets of mutant flux data. Metab Eng. 2014. doi:10.1016/j.ymben.2014.05.014;

11. Jahan N, Maeda K, Matsuoka Y, Sugimoto Y, Kurata H. Development of an accurate kinetic model for the central carbon metabolism of *Escherichia coli*. Microb Cell Fact. 2016. doi:10.1186/s12934-016-0511-x;

12. Kadir TAA, Mannan AA, Kierzek AM, McFadden J, Shimizu K. Modeling and simulation of the main metabolism in *Escherichia coli* and its several single-gene knockout mutants with experimental verification. Microb Cell Fact. 2010. doi:10.1186/1475-2859-9-88;

13. Matsuoka Y, Shimizu K. Catabolite regulation analysis of *Escherichia coli* for acetate overflow mechanism and co-consumption of multiple sugars based on systems biology approach using computer simulation. J Biotechnol. 2013. doi:10.1016/j.jbiotec.2013.06.023;

14. Kotte O, Zaugg JB, Heinemann M. Bacterial adaptation through distributed sensing of metabolic fluxes. Mol Syst Biol. 2010. doi:10.1038/msb.2010.10;

15. Soini J, Ukkonen K, Neubauer P. High cell density media for *Escherichia coli* are generally designed for aerobic cultivations - Consequences for large-scale bioprocesses and shake flask cultures. Microb Cell Fact. 2008. doi:10.1186/1475-2859-7-26;

16. Ren Q, Henes B, Fairhead M, Thöny-Meyer L. High level production of tyrosinase in recombinant *Escherichia coli*. BMC Biotechnol. 2013. doi:10.1186/1472-6750-13-18;

17. Sauer U, Lasko DR, Fiaux J, Hochuli M, Glaser R, Szyperski T, et al. Metabolic flux ratio analysis of genetic and environmental modulations of *Escherichia coli* central carbon metabolism. J Bacteriol. 1999. doi:10.1128/jb.181.21.6679-6688.1999;

18. Heldal M, Norland S, Tumyr O. X-ray microanalytic method for measurement of dry matter and elemental content of individual bacteria. Appl Environ Microbiol. 1985. doi:10.1128/aem.50.5.1251-1257.1985;

19. Jeremy EP, Ravi Radhakrishnan, Diamond SL. Steady-state kinetic modeling constrains cellular resting states and dynamic behavior. PLoS Comput Biol. 2009. doi:10.1371/journal.pcbi.1000298;

20. Nakano T, Doi T, Yoshimoto J, Doya K. A kinetic model of dopamine- and calcium-dependent striatal synaptic plasticity. PLoS Comput Biol. 2010. doi:10.1371/journal.pcbi.1000670;

21. Song CW, Kim JW, Cho IJ, Lee SY. Metabolic Engineering of *Escherichia coli* for the Production of 3-Hydroxypropionic Acid and Malonic Acid through *β*-Alanine Route. ACS Synth Biol. 2016;5: 1256–1263. doi:10.1021/acssynbio.6b00007;
