# Supplementary material for: A kinetic model of the central carbon metabolism for acrylic acid production in Escherichia coli
Source: PLoS Comput Biol. 2021 Mar 8;17(3):e1008704. doi: 10.1371/journal.pcbi.1008704 (PMC7971886; doi:10.1371/journal.pcbi.1008704)
Supplement: Attachment — Submitted filename: Review_Oliveira_PCB_2020.docx [file pcbi.1008704.s013.docx]

The topic is interesting and in the scope of PLOS Computational Biology. The question addressed by this work (identification of the optimal biosynthetic pathway and carbon source for production of acrylic acid, and of the reaction steps controlling this production) is clear, and the approach is meaningful.

Some important points should be clarified before acceptation, but I am confident all of them can be addressed by the authors in a revised version of the manuscript. The main comment is the lack of validation of a major outcome of this study, i.e. the identification of enzymes that control the production flux and should be overexpressed to improve acrylic acid production. Some methodological aspects should also be improved, as detailed below.

Major comments:

- The kinetic model used in this study was initially developed, validated and analyzed for *E. coli* grown on glucose, which is one of the condition analysed in this study. The model is thus expected to be valid in this condition. However, the authors also investigate production on glycerol, which enters into glycolysis at the level of trioses phosphate and results in a significant change of intracellular fluxes. How close is the flux distribution predicted by the model on glycerol (in absence of production) compared to the flux distribution measured on this carbon source (e.g. obtained by ^13^C-metabolic flux analysis, DOI 10.1016/j.jbiosc.2017.09.009)? This should be evaluated and briefly discussed (either in the main text – at least mentioned here – or in supplementary data). This would strengthen the predictions obtained on glycerol, and could be used to validate some methodological aspects (e.g. to evaluate the relevance of the method used to estimate unknown parameters).
- Most reactions of the production pathways are modelled as an irreversible process. However, there are many examples where product accumulation inhibits the flux, due for instance to thermodynamics constraints, and this is not taken into account in the model. For instance, the production of acetate from glucose decreases as soon as acetate concentration reaches a few millimolar (DOI 10.1038/srep42135). Consistently, in some situations, the end-products must be removed from the environment of cells to maintain a production (e.g. DOI 10.1016/s0167-7799(99)01351-7). I am not sure whether this would affect the outcome of this work or not, but I think it would be worth implementing reversible rate laws (several generic reversible rate laws are available in the literature). Similar considerations about the glycerol uptake pathways could also explain why some models were “*unable to reach a steady-state*” (lines 194, 204, 229) in some conditions, and why “*model presented issues with dihydroxyacetone accumulation*” (line 191). At the level of the production pathway, the malonyl-CoA reductase catalyzes the reaction McoaR, which is defined as reversible in Brenda and other databases. Similarly, the reaction catalysed by the beta alanine aminotransferase (BaAT) is reversible (most reactions catalyzed by aminotransferases are freely reversible, with ΔG°' ≈ 0 kJ/mol). 3-HPA dehydrogenase is also subject to product inhibition (e.g. see DOI 10.1038/s41598-017-15400-x). Please check for other reactions/enzymes, and modify the model accordingly.
- Most of the Vmax values used in the model are in a reasonable range, but some values are far too high. For instance, the Vmax of AccC is set to 0.568 mM/s (i.e. mmol/L_cytosol/second), which corresponds to 3.6 mmol/gDW/h and is in a correct range. In contrast, the Vmax of AspAT is set to 127 mM/s, which corresponds to 809 mmol/gDW/h and is much above a biologically plausible value. This might indeed be “*ideal for maximizing the production yields*” (lines 236-237) but is hardly conceivable in practice. Some of the tested Vmax values are even higher (e.g. for some simulations the Vmax of GlyD was set to 4300 mM/s, which corresponds to 27400 mmol/gDW/h!!, S1 appendix). Parameters values should be kept within a biologically feasible range. The constraints should thus be adapted accordingly during model construction and optimization. This might be related to the enzyme concentration assumed for all reactions, which was 100 mM. This corresponds to roughly 10^8^ copies of proteins per *E. coli* cell, i.e. much higher than the concentration of the most abundant enzymes. This value is two orders of magnitude higher than the protein expression limit estimated in yeast (DOI 10.1091/mbc.E15-07-0512) and about two orders of magnitude higher than the entire proteome of an *E. coli* cell (estimated at 3.10^6^ copies per cell, bionumbers ID 100088).
- For batch simulations, the substrate (glucose or glycerol) is consumed at a constant rate, and the product accumulates at a constant rate. A similar behaviour is observed for all simulated profiles. This should not be the case in batch condition (due to exponential growth of *E. coli*). These artifactual simulated profiles emerge from defining biomass concentration as constant. When having a look at the models, we can see that growth is indeed simulated, but there is no accumulation of biomass (which is not even defined as a specie). Hence, the authors cannot state that “*a batch version of the model was used for the optimisation*” (line 395 and elsewhere). This should be corrected (by defining biomass as a specie and taking into account its concentration in the changes of extracellular species – substrates and products -).
- There is no validation of a major outcome of this study, i.e. the identification of reactions that control AA production. This is currently lacking and would greatly improve the manuscript. In my opinion, it is important to validate (at least qualitatively) that increasing the rates of the proposed controlling reactions actually translates into an increased production of 3-HP or AA. This could be done by quantifying production in strains overexpressing (for instance on a plasmid) enzymes that catalyse the identified reactions.
- All models are available at <https://cutt.ly/aaKineticModels>, thanks for sharing these models with the community. However, this address appears to refer to an URL shortener service, which cannot guarantee their long-term availability. Please, provide models via dedicated repositories (such as biomodels database) to ensure long-term availability.

Minor comments:

- The models and the general approach used in this work are not described at all in the results section (though all relevant information is given in the methods section and in supplementary information). It would be worth adding a paragraph at the beginning of the results to briefly describe the models (structure, construction approach, limitations…) and refer to the methods section for more detailed information. Lines 277-280 could be moved to this paragraph. This would make the manuscript easier to read.
- Please check the figures (and tables) numbering in the text (I think most of them are wrong, e.g. Fig 2 in the text line 313 should not refer to simulation results but to the production pathways, which are shown in Fig 1)
- Line 139: “the behaviour analysis of the model…” Which model? Glu-Mcoa or Gly-Mcoa?
- Line 184: Reference to Table 5 should be Table 3. The results obtained for the beta-alanine pathway are not so “*promising*” (line 183) since this pathway has by far the lowest predicted and measured titer (as shown in table 3)
- Line 261: “with the useD of glycerol” => “with the use of glycerol”
- GluD is defined as a reversible reaction (equation 9), but the rate law corresponds to an irreversible reaction (table 5). Please correct.
- Equation (13) which describes the 3-hydroxypropionaldehyde dehydrogenase (3hpaD) reaction: the product is 3-HP, not 3-HPA. Please correct.
